# Nogo Receptor 1 Limits Tactile Task Performance Independent of Basal Anatomical Plasticity

**DOI:** 10.1371/journal.pone.0112678

**Published:** 2014-11-11

**Authors:** Jennifer I. Park, Michael G. Frantz, Ryan J. Kast, Katherine S. Chapman, Hilary M. Dorton, Céleste-Élise Stephany, Megan T. Arnett, David H. Herman, Aaron W. McGee

**Affiliations:** 1 Developmental Neuroscience Program, Saban Research Institute, Children's Hospital Los Angeles, Department of Pediatrics, Keck School of Medicine, University of Southern California, Los Angeles, California 90027, United States of America; 2 Section of Neurobiology, Department of Biological Sciences, University of Southern California, Los Angeles, California 90089, United States of America; University of Nebraska Medical Center, United States of America

## Abstract

The genes that govern how experience refines neural circuitry and alters synaptic structural plasticity are poorly understood. The nogo-66 receptor 1 gene (*ngr1*) is one candidate that may restrict the rate of learning as well as basal anatomical plasticity in adult cerebral cortex. To investigate if *ngr1* limits the rate of learning we tested adult *ngr1* null mice on a tactile learning task. *Ngr1* mutants display greater overall performance despite a normal rate of improvement on the gap-cross assay, a whisker-dependent learning paradigm. To determine if *ngr1* restricts basal anatomical plasticity in the associated sensory cortex, we repeatedly imaged dendritic spines and axonal varicosities of both constitutive and conditional adult *ngr1* mutant mice in somatosensory barrel cortex for two weeks through cranial windows with two-photon chronic *in vivo* imaging. Neither constant nor acute deletion of *ngr1* affected turnover or stability of dendritic spines or axonal boutons. The improved performance on the gap-cross task is not attributable to greater motor coordination, as *ngr1* mutant mice possess a mild deficit in overall performance and a normal learning rate on the rotarod, a motor task. Mice lacking *ngr1* also exhibit normal induction of tone-associated fear conditioning yet accelerated fear extinction and impaired consolidation. Thus, *ngr1* alters tactile and motor task performance but does not appear to limit the rate of tactile or motor learning, nor determine the low set point for synaptic turnover in sensory cortex.

## Introduction

Experience sculpts the function and synaptic connectivity of neural circuitry, yet both functional and anatomical plasticity diminish as development concludes [1], [2]. In adult mice, sensory adaptation, motor learning, and fear conditioning are associated with elevated cortical spine dynamics [3]–[10]. The genes and mechanisms that govern the magnitude of this anatomical cortical plasticity in the developing and mature brain in response to experience are poorly understood.

The nogo-66 receptor 1 gene (*ngr1*) is one candidate that may restrict anatomical plasticity within adult cortical circuitry [11]. NgR1 is a neuronal receptor for a number of disparate ligands that inhibit neurite outgrowth *in vitro* [12], [13], and suppression of NgR1 expression by primary embryonic hippocampal neurons increases spine density *in vitro* [14]. NgR1 is also required to close a ‘critical period’ for developmental visual plasticity [15]. A recent study reported that *ngr1* mutant mice display a faster rate of improvement on the rotarod, an assay of motor coordination, as well as dramatically increased basal spine dynamics and stability in both sensory and motor cortex [11]. Thus, NgR1 has been proposed to be a critical molecular determinant gating the transition from rapid anatomical plasticity in adolescence to lower dendritic spine dynamics in adulthood that restricts the effects of experience on cortical anatomy.

The gap crossing (GC) learning paradigm is a prime example of a distance detection/object localization task [16]–[18]. In this task, animals are placed on an elevated starting (“home”) platform in a light-tight enclosure and explore the dark environment using their whiskers to locate a target platform placed at user-defined distance from the home platform. At short distances, mice perform the task by contacting the target platform with their whiskers and nose, activating whiskers as well as touch receptors in the skin around the nose. At longer distances they must solely rely on their whiskers for tactile information [19]. Successful task acquisition requires intact somatosensory ‘barrel’ cortex. Mice improve their performance on this task with experience; this learning yields a greater percentage of successful crossings of a given distance in successive sessions of trials.

Here we tested the role of NgR1 as a critical gate to both experience-dependent learning and anatomical plasticity in sensory cortex. We compared the overall performance and rate of learning across sessions with this whisker-dependent learning task and basal anatomical plasticity in barrel cortex with chronic two-photon *in vivo* imaging by adult *ngr1*−/− mice and wild-type (WT) controls. Mice lacking *ngr1* displayed typical improvement across sessions despite better initial performance. In contrast to a preceding study [11], we observed that the basal dynamics of both dendritic spines and axonal boutons were indistinguishable between *ngr1*−/− mice and controls. *Ngr1*−/− mice also exhibited a minor deficit in performance yet normal improvement on the rotarod, as well as normal tone-associated fear conditioning. Interestingly, *ngr1*−/− mice displayed accelerated extinction coupled with reduced consolidation. Thus, we conclude that *ngr1* contributes to performance on both sensory and motor tasks but does not restrict either the rate of learning or basal synaptic turnover in the sensory cortex.

## Materials and Methods

### Mice

The constitutive *ngr1*−/− and conditional *ngr1flx/flx* strains have been described previously [20], [21]. The *ngr1−/−* strain was F8 and the ngr1flx/flx strain was F6 when these mice were re-derived (Jackson Labs). Subsequently, the ngr1flx/flx strain was backcrossed onto the C57Bl6 background to F8+. Each line was then backcrossed against C57Bl6 Thy1-EGFP-M transgenic mice obtained from a commercial vendor [22] (The Jackson Laboratory). Mice were group housed with same-sex littermates and food and water were available *ad libitum*. These *ngr1* strains are identical to those used in two preceding studies examining cortical spine dynamics [11], [23].

Mice were maintained and all experiments conducted according to protocols approved by the Children's Hospital Los Angeles Institutional Animal Care and Use Committee. Mice were anesthetized by isoflurane inhalation and euthanized by carbon dioxide asphyxiation in accordance with approved protocols. The Children's Hospital Los Angeles Institutional Animal Care and Use Committee specifically approved this study. Protocol number 264-12.

### The Gap Cross Assay

The gap cross assay was performed with a custom-built robot (D.H. Herman, manuscript in preparation). In brief, the gap cross assay system is a closed-loop robotic environment with motor controlled units and sensing elements. The mouse behaves upon raised platforms driven by independent linear actuators. The platforms are equipped with servo-motor doors and positional sensors. Data acquisition and control algorithms are both executed online for real-time dynamic control and offline for more advanced analysis.

Independent linear actuators move the Plexiglass platforms to generate a range of gap-distances from nose (<5 cm) to whisker (5–7 cm) distances. To monitor the rodent motion four IR motion sensors are at the back and edge of each platform. Near the edge of each platform are servo-controlled doors that prevent exploratory behavior during repositioning of the platforms. The linear motors, servos, and motion sensors are USB controlled through microcontroller boards (Arduino Mega 2560 and the Quadstepper Motor Driver) that communicate with a quad-core CPU.

Motor positions are processed on a quad-core CPU using the Arduino and Matlab programming environments. Platform position, door status (open/closed) and feeders are real-time controlled using the Arduino C-based development environment (ADE). Motion sensor data are continuously acquired and pre-processed within ADE and are visualized and stored in real time within Matlab via serial communication. Specifically, sensor activity are encoded as behavioral performance metrics: success/failed crossing events, 1) successful: animal approaches the gap and crosses to back of target platform; or 2) failed: animal approaches the gap and then retreats to back of home platform. This information is computed in real-time.

To control the positional and door motors, the GCS employs a closed-loop finite state machine algorithm (D.H. Herman, unpublished observations). Animal behaviors are segmented into interactive events at the gap. Consequently, the system is structured as a two state machine: Exploration and Adjustment. During Exploration, the motors are disabled and the system continuously acquires behavioral data via motion sensors. During Adjustment, the doors close to halt exploration, and the motors reposition the platforms for the next exploration phase as determined by the programmed protocol. Transitions between the two states are triggered by behavioral events (i.e. successful/failed gap-crossing).

Male littermates were group housed with food and water available *ad libitum*. The mice were 10–12 weeks old at the start of the task. Animals were handled for 10 minutes a day for one week prior to beginning the task. The day before training began, mice were habituated to the gap cross apparatus by placing each mouse in the chamber with background white noise for 20 minutes in white light immediately followed by 20 minutes in the dark. A bridge was placed over the gap to prevent exploration of the gap and gap crossing behavior during habituation. Mice were subsequently trained once per day. Each session lasted 20 minutes or 20 successful crosses, whichever came first.

### Cranial windows

Male and female c57/Bl6 EGFP-M transgenic mice expressing green fluorescent protein in a sparse population of cortical layer 5 pyramidal neurons (transgenic line M; Jackson Laboratories) were used. WT, *ngr1−/−*, and *ngr1flx/flx;Cre-ER* mice received cranial windows after P60 and imaging was initiated 4 or more weeks later. Mice were anaesthetized with isoflurane and administered dexamethasone (4 µg/g body weight) subcutaneously. Body temperature was maintained with a biofeedback heatpad (Physitemp). Cranial windows were implanted as previously described, with a minor modifications [24]. A circular region of the skull over barrel cortex or visual cortex was removed without perturbing the underlying dura. A 2.5 mm diameter #1 thickness cover glass (Bellco) was placed on the dura, affixed with cyanoacrylate (Krazyglue), and sealed with dental acrylic. A small aluminum bar with tapped screw holes was embedded into the acrylic to stabilize the animal for subsequent imaging sessions. Animals received buprenorphine (0.1 µg/g body weight) and baytril (1∶1000) in water post-surgery. Their water was also supplemented with carprofen (1∶2000) throughout the imaging series. Animals were given at least 2 weeks to recover before imaging as cranial windows that were optically clear at 2 weeks were likely to remain clear for the duration of the imaging series. In these experiments, mice were imaged over 4-day intervals beginning 4 weeks after implanting cranial windows.

### Optical Imaging of Intrinsic Signals

Imaging was performed as described previously [25]–[27]. Mice were administered chlorprothixene (1 µg/g body weight) and isoflurane anesthesia was maintained near 1%. To visualize whisker-evoked changes in intrinsic signals in S1 barrel cortex, a single whisker (e.g. C2) contralateral to the cranial window was deflected approximately 15 degrees with a 3 Hz sinusoidal pulse train for 3 s every 20 s using a piezoelectric actuator controlled by a function generator (GW Instek). This was repeated 35 times per run.

Green light (530 nm±30 nm) was used to visualize cerebral vasculature and red light (620 nm±20 nm) to image intrinsic signals. The imaging plane was focused ∼200–400 µm below the cortical surface. Images were acquired at 10 Hz at 1024×1024 pixels per image at 12-bit depth with a high-speed camera (Dalsa 1M60) and custom acquisition and analysis software (C++ and Matlab). Collected images were spatially binned before the response at the stimulus frequency was extracted from a complete time series for each pixel by Fourier analysis.

### Chronic *In Vivo* Two-Photon Imaging

All imaging was conducted blind to the genotype. Mice were imaged at least 4 weeks after implanting the cranial window and the average age of mice was similar between genotypes at start of imaging (WT, P113–150, Average P130; *ngr1−/−*, P130–173, average P140; *ngr1flx/flx;Cre-ER* P104). Animals were anaesthetized with isoflurane and body temperature was maintained with a biofeedback heat pad (Physitemp). Images were acquired with a modified Movable Objective Microscope (MOM) (Sutter Instruments) and 40× 1.0 NA water immersion objective (Zeiss) using scanimage software (MatLab) [28]. The light source is a Ti:sapphire tunable laser (Chameleon Ultra II, Coherent) operating at 910 nm. Imaging typically required less than 50 mW of power. The identity of L5 neurons was confirmed by measuring the depth at which the cell body resided. Image stacks consisted of sections (512×512 pixels) collected in 1 µm steps. Low-magnification images (0.56 µm/pixel) were taken to visualize dendritic arbors and branching patterns. These guided the high-magnification images (0.14 µm/pixel) collected for spine analysis. Care was taken to maintain the same level of fluorescence across imaging intervals. Animals were imaged every 4 days. Imaging sessions lasted no more than 2 hours. All images of neuronal structures presented in this study are best projections from z-stacks after linear contrast adjustment (Photoshop software, Adobe).

### Image Analysis for Dendritic Spines

Image analysis was done following published guidelines [24]. All analysis was done blind to genotype using ImageJ (NIH). Clearly defined protrusions from the dendrite present in the first imaging interval were labeled. In image stacks from subsequent imaging sessions, experimenters determined whether a labeled spine was still present or not, and checked for the appearance of new spines. Newly added spines were also tracked throughout subsequent imaging sessions. Series of image stacks for each field were analyzed by two experimenters independently. In the case of a discrepancy between the two sets of analysis, a third experimenter repeated the analysis.

### Image Analysis for axonal boutons

Boutons were identified and image analysis performed following published guidelines [21], [29]. Boutons were defined as new if they were three times brighter than the surrounding axon and lost if their fluorescence decreased to lower than 1.3 times the surrounding axon. All analysis was done blind to genotype using ImageJ (NIH). Boutons present in the first imaging session were labeled. In image stacks from subsequent imaging sessions, experimenters determined whether a labeled bouton was still present or not, and checked for the appearance of new boutons. Newly added boutons were also tracked throughout subsequent imaging sessions. An experimenter blind to genotype analyzed the image stack series for each field. In the case of a discrepancy between the two sets of analysis, a third experimenter repeated the analysis.

### Image Presentation

Images in synaptic structures are ‘best projections’, a montage of the best focal plane for each spine or region within a stack of images in the *z*-plane. This montage has received only linear contrast adjustment, save for the high magnification images in panel 3B that also were filtered with a 3-pixel radius Gaussian blur for presentation.

### Tamoxifen Injections

Tamoxifen treatment was performed as previously described [11], [21]. In brief, tamoxifen (Sigma, T5648) was solubilized in corn oil at 10 mg/ml. Once a day for three consecutive days mice received an intraperitoneal injection of tamoxifen at concentration of 100 mg/kg (1 mg/10 g body mass).

### Rotarod

Adult female mice (3–5 months of age) were trained on an accelerating, rotating drum as previously described [11], [20], [30]. Mice were habituated to the static rod for one 10-minute session per day for three days prior to training. Mice were given three trials per day for five consecutive days. The rotating drum was accelerated from 4 to 44 rpm over a period of 200 seconds (the same ramp speed as Akbik et al., 2013) and the latency to fall off the rod was recorded. Each trial was separated by at least 30 minutes. Consistent with numerous published protocols, a passive rotation counted as a failure. At the conclusion of the five days of training at the higher acceleration rate, mice were tested on the sixth day using a slower ramp speed corresponding to 4–40 rpm over 5 minutes. Latency to fall was averaged over three trials separated by at least a 30-minute interval between trials [11], [20], [30], [31].

### Tone-associated Fear Conditioning and Extinction

Fear conditioning and extinction were performed as described previously [11], [15], [31]. Conditioning and extinction were performed in a sound-dampened chamber containing a tone generator and light-emitting diode (LED) light source. The conditioning chamber, context A, was plexiglass with a metal rail floor (MedAssociates) and was cleaned before and after each session with 70% ethanol. On day 1, adult female WT and *ngr1−/−* mice (3–5 months of age) that were co-housed were first placed in context A for 15′ to acclimate. A few hours later, mice were again placed in context A and conditioned with 5 pairings of the conditioned stimulus, a 30 s 80 dB 2 kHz tone, with a 1 s scrambled foot shock (0.6 mA or 0.3 mA, MedAssociates) that coterminated with the tone. A separate auditory cue, 80 dB white noise, was presented after each conditioning stimulus. The extinction chamber, context B, was a plexiglass box with a removable plastic floor and was cleaned before and after each session with Process NPD (Steris Life Sciences). On day 2 and 3, 24-hours and 48-hours after conditioning, respectively, mice were placed in context B and received 12 presentations of the conditioned stimulus and 4 presentations of the white noise stimulus, each lasting 30 s. Percent time freezing was determined with FreezeFrame video analysis software (Coulborn instruments).

### Immunohistochemistry

Staining for myelin basic protein (MBP) and Nogo-A were performed as described previously [15]. At postnatal day 24 or 40 (P24, P40), mice were deeply anesthetized with Ketamine HCl (200 mg/Kg, Phoenix pharmaceuticals)/ Xylazine (20 mg/Kg, Lloyd Laboratories) and transcardially perfused with phosphate buffered saline (PBS; ChemCruz SC-362299) followed by a buffered 4% paraformaldehyde (PFA)/PBS (Acros Organics 416780030). Brains post-fixed overnight in 4% PFA/PBS. Free-floating 40 µm sections were cut on a vibrating microtome (Leica VT 1000S) and preserved in PBS containing 0.05% sodium azide (Sigma-Aldrich S8032).

Coronal sections containing visual cortex or somatosensory cortex were washed in Tris-Buffered Saline (TBS, 50 mM Tris-HCl, 150 mM NaCl, pH 7.4) (3×5 minutes) and then incubated in 100 mM sodium citrate buffer pH 4.5 (Sigma-Aldrich S1804) at 95C for 10 minutes for antigen retrieval. Sections were allowed to cool to room temperature and then washed in TBS (3×10 minutes). Sections were incubated in blocking solution, 3% normal horse serum (NHS; Vector Laboratories S-2000) in TBS containing 0.1% Triton X-100 (Sigma-Aldrich T9284), for 1 hour at room temperature (RT). The primary antibody mouse αmyelin basic protein (MBP) (Covance, SMI-94R) was diluted in blocking solution to 1 µg/mL. Sections incubated in primary antibody overnight at 4°C. After repeated washing in TBS-T (3×10 min), sections were incubated in secondary antibody, Alexa 488-conjugated goat αmouse (Jackson Immuno Research) 1∶200 in blocking solution, for 1 hour at RT. After a final series of washes (3×10 min in TBS-T, 1×10 min in TBS), sections were mounted onto SuperFrost Plus slides (Fischer) with Fluoromount G containing 4′,6-diamidino-2-phenylindole (DAPI) (Electron Microscopy Science).

### Analysis of MBP distribution

Images from coronal sections stained with αMBP/Alexa488 were captured with a BX-51 microscope, 5× 0.4 NA objective and 12-bit monochrome camera (Retiga EX, QImaging). DAPI staining was utilized to identify visual cortex and somatosensory cortex prior to capturing images of MBP density. Two images were required to span the distance from the subcortical white matter to the pial surface. Images were merged with the software Photoshop following linear contrast adjustment and scaled down to 100 pixels spanning cortex. Each pixel was normalized to the intensity of the deepest pixel in subcortical white matter. Data points are the average of at least four hemispheres from two or more sections from each of two or three animals for each age.

### Statistics

Statistical comparisons were performed with Prism software (GraphPad). All comparisons of spine and bouton gains and losses were made with the Mann-Whitney test (unless otherwise noted) as the *n* for these experiments is too small to confirm a normal distribution. Turnover ratios for *ngr1*flx/flx Cre-ER mice were compared by repeated measures (RM) one-way ANOVA with Dunn's correction for multiple comparisons. Gap cross performance and improvement, motor performance and improvement, and fear extinction, were compared between genotypes by two-way ANOVA with Bonferroni correction for multiple comparisons and unpaired two-tailed t-test with Welch's correction for pairwise comparisons.

## Results

**Figure 1:**
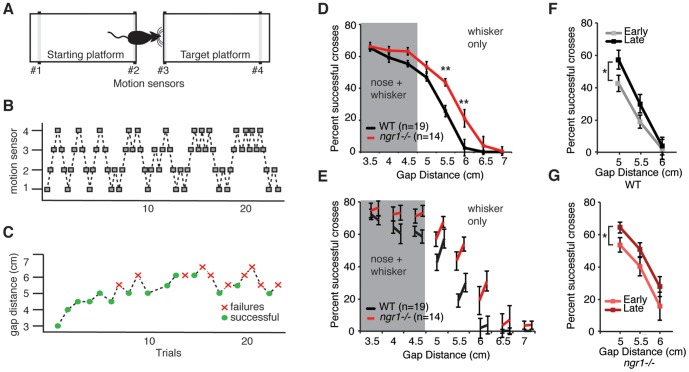
Mice lacking *ngr1* perform better on the gap cross assay but display normal tactile learning across sessions. (A) A schematic of the gap cross assay. The movement of a mouse from the starting, or ‘home’, platform to the target platform across a given gap distance is detected with motion sensors positioned at the back and edge of each platform. (B) Activation of each sensor (grey box) indicates the position of the mouse. (C) Successful crosses are defined as the movement of the mouse from the starting platform to the target platform (green circles). Failures are defined as trials in which the mouse approaches the edge of the home or target platform and returns to the back of the home platform (red crosses). (D) *ngr1−/−* mice cross ‘whisker’ distances at a significantly higher success rate (WT, n = 19; *ngr1−/−*, n = 14; p>.01 for distances 5.5 and 6 cm; p>.32 for distances 3.5 and 4 cm, two-way ANOVA). This greater success rate is most significant at longer distances, 5.5 cm and 6 cm (**, p>.01 with Bonferroni correction for multiple comparisons). (E) Despite better overall performance, the percent improvement for a given gap distance from the first 4 sessions (left value for each distance) to the second 4 sessions (right value for each distance) is similar for WT and *ngr1−/−* mice for a given gap distance. (F) WT mice improve with experience at ‘whisker’ gap distances (WT, n = 19, *, p<.05, two-way repeated measures ANOVA) from the first 4 sessions (Early, grey line) to the second 4 sessions (Late, black line). (G) *Ngr1* mutant mice improve with experience at ‘whisker only’ gap distances (*ngr1−/−*, n = 14, *, p<.05, two-way repeated measures ANOVA) from the first 4 sessions (Early, pink line) to the second 4 sessions (Late, red line).

To investigate if *ngr1* limits the rate of sensory or motor learning, we examined the improvement in performance of *ngr1−/−* and WT mice on both sensory and motor learning tasks. First, we tested *ngr1−/−* and WT mice on a validated test of tactile learning, the gap cross assay. This assay requires both tactile stimulation of the whiskers and intact somatosensory cortex [16]–[19]. In these experiments, we relied on the natural exploratory behaviour of mice to perform the task. To minimize variation associated with different responses to motivation, performance was not rewarded by delivery of an appetitive food pellet with or without food restriction. Mice were acclimated to the device and then tested at a range of distances spanning 3.0 to 6.0 cm for 20 successful trials or a maximum of 20 minutes per day for 8 consecutive days. All sessions began with a trial at 3.0 cm, the shortest distance tested. Position of the mouse was tracked with motion sensors placed at the back and near the edge of each platform (Fig. 1A). As a mouse traversed the platform, these sensors record its progressive position (Fig. 1B). A successful trial was identified as any trial in which the mouse successfully crossed the gap between the home and target platforms and activated the motion sensor at the back of the target platform (Fig. 1C). A failed attempt was defined as an attempt in which the mouse explored the edge of the home platform and returned to the back of the platform (Fig. 1C). Thereafter, the next distance was determined with a learning algorithm that randomly chose the distance from a Gaussian distribution centered a gap distance 0.5 cm longer than the previous distance if the preceding trial were successful, and a gap distance 0.5 cm shorter if the preceding trial were a failure (D.H. Herman, manuscript in preparation). This approach decreases the predictability of the subsequent gap distance relative to a ‘laddering’ learning paradigm in which the next distance increased or decreased by a set distance depending on the success or failure in the preceding trial.

WT and *ngr1−/−* mice displayed a similar high probability to cross at distances less than 4.5 cm (Fig. 1D). At these ‘nose-distances’, mice are able to detect the target platform by touching it with their nose as well as whiskers (nose+whisker, Fig. 1D). In contrast, mice acquire tactile information exclusively with their whiskers at longer distances: 5.0, 5.5 and 6.0 cm. At these ‘whisker-distances’, overall performance declines with increasing gap distance (whisker only, Fig. 1D). However, overall performance was significantly higher for *ngr1−/−* mice at whisker-distances but not the closer ‘nose-distances’ (Fig. 1D) (WT, n = 19; *ngr1−/−*, n = 14; p>.01 for distances 5.5 and 6 cm; p>.32 for distances 3.5 and 4 cm, two-way ANOVA). To assess improvement in performance with experience, we compared the probability of successful crossing between the first four sessions (1–4) and the subsequent four sessions (5–8) at all gap distances tested. At the shorter ‘nose-distances’, sensory information is more definitive and mice exhibit little improvement in performance between the first four sessions and subsequent four sessions (Fig. 1E) (WT, p>.42; *ngr1−/−*, p>.79; two-way RM-ANOVA). However, at the longer ‘whisker-distances’, both WT and *ngr1−/−* mice improve with experience (Fig. 1E–G) (WT, p<.01; *ngr1−/−*, p<.05; two-way RM-ANOVA). The percentage of improvement by WT and *ngr1−/−* mice was greatest at the shortest of the three ‘whisker-distances’ (Fig. 1F,G), and the two genotypes exhibited similar improvement across these three distances (Fig. 1G) (Two-way ANOVA, p>.75; WT, n = 19; *ngr1−/−*, n  =  14; p>.97 for each corrected pairwise comparison for distances 5.0, 5.5, and 6.0 cm). In addition, although *ngr1−/−* mice are reported to be slightly hypoactive in an open field test [20], WT and *ngr1−/−* mice performed a similar number of total trials (WT, 142.1±9.9 trials; *ngr1−/−*, 165.0±10.5 trials; p>.18). Thus, *ngr1−/−* mice display greater overall performance but not an evident difference from WT mice in the rate of improvement at any given gap distance across sessions.

**Figure 2:**
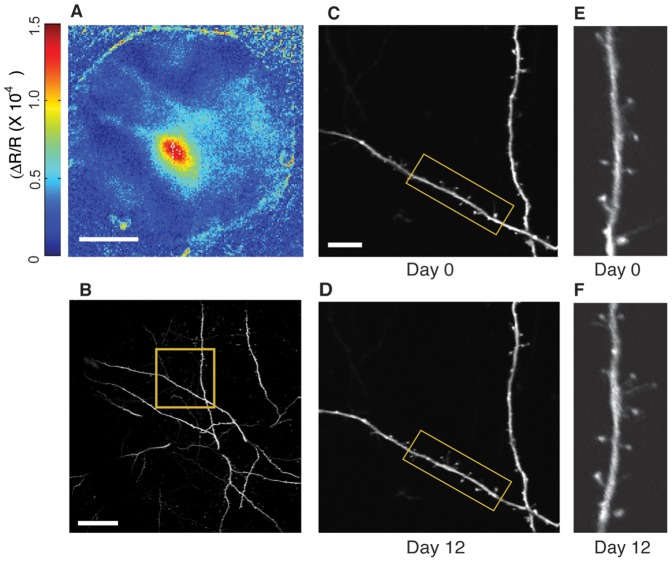
Cranial windows are properly positioned over S1 barrel cortex and are a stable preparation for imaging cortical spine dynamics. (A) An example of optical imaging of intrinsic signals reveals the cortical region responsive to stimulation of the C2 whisker. Scale bar = 0.5 mm (B) Apical dendrites of layer V neurons in the boxed region (yellow) are shown at higher magnification in panels C and D. Scale bar = 50 µm (C) Higher magnification images of the boxed region (yellow) in panel B at day 0 (D) Higher magnification images of the boxed region (yellow) in panel B at day 12 (E) Higher magnification image of the boxed region in panel C on day 0. (F) Higher magnification image of the boxed region in panel D on day 12.

**Figure 3:**
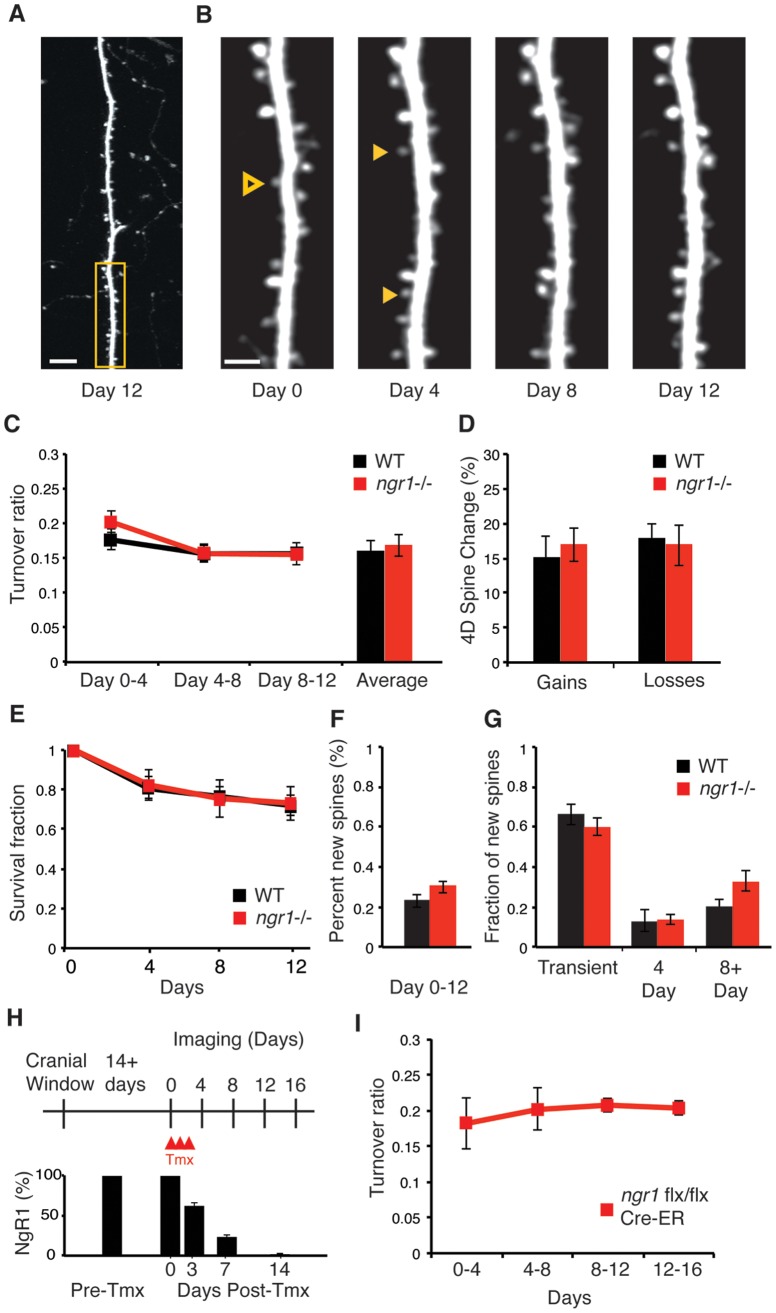
Dendritic spine turnover and stability are normal in *ngr1*−/− mice. (A) Repeated *in vivo* two-photon imaging through cranial windows in EGFP-M transgenic mice reveals the turnover and stability of dendritic spines on the apical dendrites of layer V pyramidal neurons in S1 barrel cortex. Scale bar = 10 µm. The boxed region (yellow) is shown at higher magnification in panel B. (B) Dendritic spines were imaged every four days for twelve days. Solid arrowheads (yellow) are examples of spine gains. Outlined arrowheads (yellow) are examples of spines lost. Scale bar = 2 µm. (C) The turnover of dendritic spines every four days in WT (n = 5; 1512 spines) and *ngr1*−/− mice (n = 4; 1106 spines) is similar across 4-day intervals (p>0.4). The average across all sessions is also comparable (p>0.9). (D) The average percent of spines gained and lost is similar between WT (n = 5) and *ngr1−/−* mice (n = 4) (gained p>0.2; lost p>0.9). (E) The survival fraction of spines present on day 0 re-examined at days 4, 8, and 12 is nearly identical (p>0.8) (F) The percent of new spines present on day 12 is similar between WT and *ngr1−/−* mice. (p>0.2) (G) The fraction of new spines appearing on day 4 that are transient (p>0.3), surviving less than 4 days, those lasting less than 8 days (present only on day 4 and 8) (p>0.8), and persistent spines surviving more than 8 days (p>0.1) are similar between WT and *ngr1−/−* mice. (H) Timeline of acute deletion of *ngr1* in *ngr1flx/flx;Cre-ER* mice following tamoxifen injection and imaging schedule as NgR1 protein levels decline. (I) Basal cortical spine dynamics in S1 barrel cortex are unaffected by acute deletion of *ngr1*. The turnover ratio does not change with the decline or absence of NgR1 protein (n = 3, 6 neurons, 1083 spines) (p>0.9).

**Figure 4:**
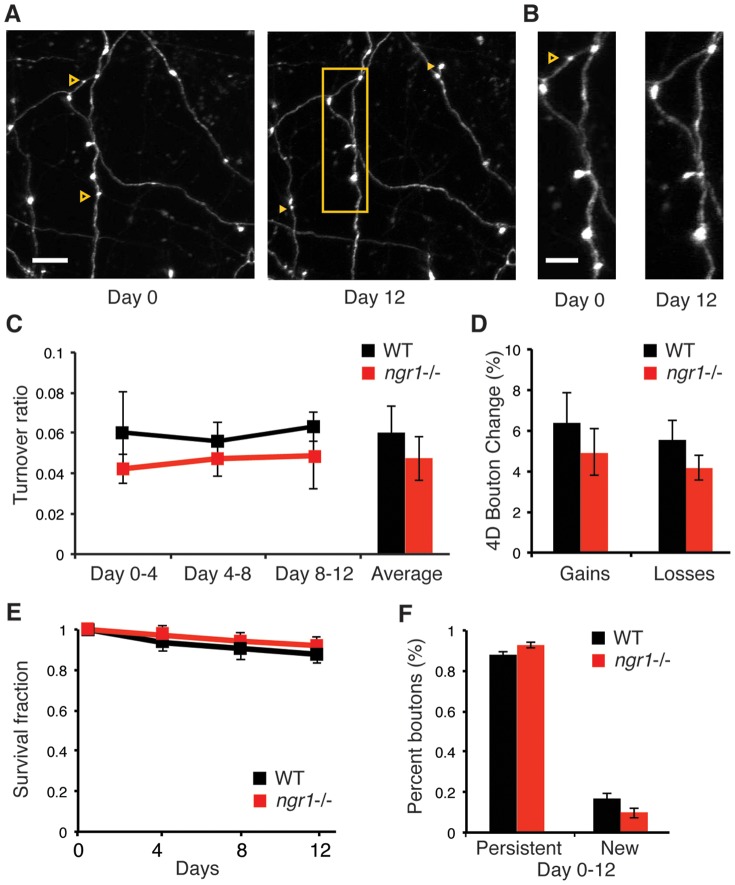
Axonal bouton turnover and stability are normal in *ngr1*−/− mice. (A) Examples of axons imaged repeatedly by repeated *in vivo* two-photon microscopy through cranial windows. Solid arrowheads (yellow) are examples of new boutons. Outlined arrowheads (yellow) are examples of boutons lost. Scale bar = 10 µm (B) Higher magnification of the boxed region (yellow) in panel A. Scale bar = 5 µm. (C) The turnover of axonal boutons every four days in WT (n = 4, 424 boutons) and *ngr1*−/− mice (n = 5, 749 boutons) is similar across 4-day intervals in S1 barrel cortex (p>0.2). (D) The average percent of axonal boutons gained and lost is similar between WT and *ngr1−/−* mice (gained p>0.5; lost p>0.6). (E) The survival fraction of boutons present on day 0 is similar at days 4, 8, and 12. (F) The percent of persistent boutons (p>0.1) and new boutons (p>0.3) present on day 12 is comparable between WT and *ngr1−/−* mice.

A preceding study reported that basal dynamics and stability of dendritic spines and axonal boutons were dramatically elevated in *ngr1−/−* mice [11]. To determine if the better performance on the gap cross performance correlated with greater basal synaptic structural plasticity, we imaged these synaptic structures in WT and *ngr1*−/− mice harboring the EGFP-M transgene in layer I of somatosensory (S1) barrel cortex with chronic *in vivo* two-photon microscopy (Figures 2–4). To check if the cranial windows were properly positioned, we determined the functional representation of the C2 barrel with intrinsic signal imaging in a subset of mice [25] (Fig. 2A). The C2 barrel was near the center of the window, consistent with location of the dendritic spines imaged in our study in S1 barrel cortex. We repeatedly imaged the apical dendrite of individual layer V neurons over 12 days (Fig. 2B–F). As the EGFP-M transgene provides sparse expression of GFP in S1 (Fig. 2B–D), we were able to obtain high quality images across multiple 4-day intervals. These images stacks were sufficient to discern not only large mushroom spines but also thinner and often transient thin spines on these dendrites (Fig. 2E,F).

Spine dynamics and stability were indistinguishable between WT and *ngr1*−/− mice. We imaged the same population of spines in 4 sessions over 12 days (WT, 1512 spines, 10 neurons, n = 5 mice; *ngr1*−/−, 1106 spines, 8 neurons, n = 4 mice) (Fig. 3A,B). The average turnover ratio was almost identical between genotypes (WT 16.3%±1.6% versus *ngr*−/− 17.1%±1.5%, p>.96) (Fig. 3C). Turnover was not significantly different between 4-day intervals for WT or *ngr1*−/− mice (p>0.9) nor between genotypes for a specified interval (p>0.4) (Fig. 3C). The average percentage of spines gained and lost every 4 days was also similar (gained, WT 15.2%±1.5% versus *ngr1*−/− 18.0%±1.1%, p>.21; lost WT 16.9%±1.2% versus *ngr1*−/− 16.9%±1.5%, p>.99, n = 5 WT and 4 *ngr1*−/− mice) (Fig. 3D). We examined both WT and *ngr1*−/− mice near P140, near the center the age range (P45–P180) for which the spine turnover in *ngr1*−/− mice has been reported to be more than double the rate of WT mice [11].

We measured the survival fraction of spines present at the first imaging session (Day 0) at three subsequent 4-day intervals to determine the stability of the overall population of spines imaged (Fig. 3E). The percentage of spines imaged on Day 0 that were present on Day 4, Day 8, and Day 12, is identical between WT and *ngr1*−/− mice (day 12, WT 72.3%±2.7% versus *ngr1*−/− 73.6%±4.6%, p>.82) (Fig. 3E,F). These rates for spine turnover and stability are consistent with previous measurements from S1 for WT mice 3–4 months of age, the same ages of mice examined here [1], [8], [32].

A subpopulation of ‘transient’ spines are continuously appearing and disappearing on excitatory neurons. These spines have a survival time constant of approximately 8 days [1]. We tracked the survival of spines that appeared during Day 4 (imaging session 2) over the following two imaging sessions on Day 8 and Day 12. The percentage of transient spines surviving less than 4 days and spines present for less than 8 days did not differ between genotypes (transient, WT 65.0%±1.5% versus *ngr1*−/− 59.5%±4.4%, p>.39; 4 Day, WT 13.4%±5.6% versus *ngr1*−/− 13.3%±2.2%, p>.87). However, there was a trend towards greater persistence for new spines in *ngr1*−/− mice (8+ Day, WT 20.9%±2.8% versus *ngr1*−/− 32.9%±5.2%, p>.11), but this difference did not reach statistical significance (Fig. 3G). A previous study reported that the 14-day stability of spines in *ngr1*−/− mice was quadruple that of WT mice [11]. However, we observe that *ngr1*−/− mice display basal spine formation, spine retraction, and new spine stability in barrel cortex that are indistinguishable from WT mice.

Next, as suppressing NgR1 expression in primary cultures of hippocampal neurons alters spine dynamics *in vitro* [14], we employed a conditionally targeted allele of *ngr1*, *ngr1*flx, to test if acute deletion of *ngr1* affects cortical spine dynamics *in vivo*. We examined *ngr1*flx/flx mice that also harbored a transgene that ubiquitously expresses a fusion protein of Cre recombinase and a mutant version of the estrogen receptor (Cre-ER) that is activated by the estrogen analog tamoxifen [21], [33]. We repeatedly imaged spines in S1 barrel cortex of *ngr1*flx/flx; *Cre-ER* mice immediately prior to tamoxifen treatment and then for another 4 sessions over 16 days (1083 spines, 6 neurons, 3 mice) (Fig. 3H,I). Densitometry of immunoblots for NgR1 confirms that tamoxifen treatment reduces NgR1 protein to less than a quarter of normal levels within a week and that NgR1 protein is not detectable after 2 weeks [21]. Spine turnover was nearly identical in *ngr1flx/flx*; *Cre-ER* mice across four 4-day intervals spanning the decline and absence of NgR1 (p>0.9) (Fig. 3I). Overall, cortical spine dynamics were not affected by the acute loss of NgR1.

We examined the corresponding turnover of axonal boutons in layer I of S1 barrel cortex (WT, 424 boutons, 4 mice; *ngr1*−/−, 749 boutons, 5 mice.) Consistent with previous studies, we employed morphological criteria to focus our analysis on axons from layer II/III and layer V neurons (type A3) [29] (Fig. 4A,B). These axons are readily distinguishable from type A2 axons decorated with terminaux boutons, and the larger, brighter, and more ramified putative thalamocortical type A1 axons. WT and *ngr1−/−* mice displayed a similar average turnover ratio, percentage of boutons gained, and percentage of boutons lost in each of three 4-day imaging intervals (turnover ratio, WT 6.0%±1.2% versus *ngr1*−/− 4.6%±1.0%, p>.21; gains, WT 6.5%±1.5% versus *ngr1*−/− 5.0%±1.2%, p>.56; losses, transient, WT 5.6%±1.1% versus *ngr1*−/− 4.2%±0.6%, p>.68) (Fig. 4C,D). The survival fraction of boutons over 12 days was also similar between genotypes, as was the percentage of new boutons present (persistent, WT 87.5%±1.9% versus *ngr1*−/− 92.0%±1.2%, p>.11; new, WT 16.7%±2.5% versus *ngr1*−/− 10.0%±2.5%, p>.38) (Fig. 4E,F). Overall, we observed no difference in the dynamics or stability of these axonal boutons between WT and *ngr1*−/− mice.

**Figure 5:**
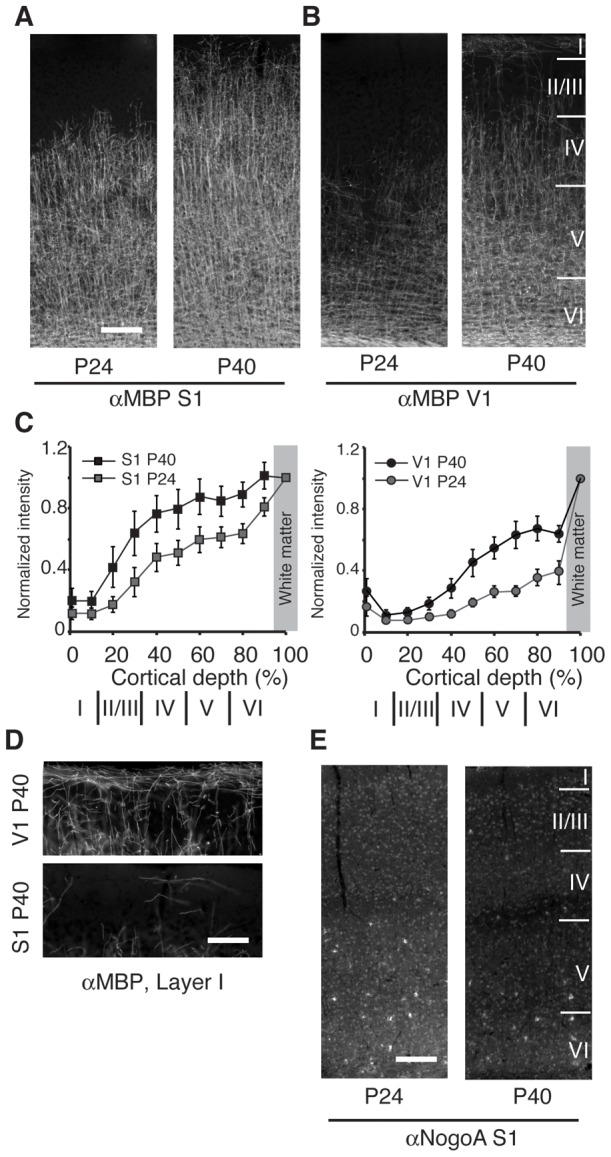
Myelination is extensive in S1 barrel cortex and does not reflect the distribution of Nogo-A. (A) Immunostaining for myelin basic protein (αMBP) of coronal sections of S1 barrel cortex reveals extensive myelination in cortical layers IV–VI at P26 when cortical spine dynamics are elevated relative to P40. Myelination increases in S1 barrel cortex by P40 to extend into layer II/III. (B) Immunostaining of V1 reveals myelination in cortical layers V–VI at P26 that extends into layer IV by P40. The approximate positions of cortical layers II/III to VI are indicated at right. The scale bar corresponds to 200 µm (C) Quantification of the relative distribution of staining intensity for myelin basic protein in S1 and V1 at P24 and P40 at increasing depths from the pial surface to the underlying white matter. Pixel intensity is normalized by the intensity of white matter (grey box). Error bars represent the standard deviation between at least 5 sections and 3 mice per group. (D) Higher magnification image of the distribution of myelinated fibers in layer I of S1 and V1 at P40. Few myelinated fibers are present in layer I of S1 relative to V1. The scale bar corresponds to 50 µm. (E) Nogo-A intensely labels the soma of putative oligodendrocytes but is also evident in cortical neurons. The pattern of expression is comparable at P26 and P40. The approximate positions of cortical layers II/III to VI are indicated at right. The scale bar corresponds to 200 µm.

Several inhibitory ligands that signal through NgR1 are associated with myelin membranes and myelination increases in layers IV and V in primary visual cortex concomitant with the close of the critical period for ocular dominance plasticity [15]. To determine if increases in cortical myelination correlate with the decline in spine dynamics in S1 observed in the transition from juvenile to adulthood [34], we examined distribution of myelinated fibers in S1 barrel cortex as revealed by immunofluorescent staining for myelin basic protein (MBP) at P26 and P40. Myelination is extensive in S1 at P26 and resembles V1 at P40 (Fig. 5A–C). The distribution and extent of myelination in S1 increases by P40 with myelinated fibers extending into layer II/III. However, unlike V1 at P40, myelination remains sparse in layer I, the location of spines we imaged with *in vivo* two-photon microscopy (Fig. 4D). In addition, we examined the pattern of Nogo-A expression in S1 cortex. The distribution of Nogo-A is distinct from myelination. As previously reported, we observe that Nogo-A is prominent in the soma of putative oligodendrocytes but is also evident in the soma of most neurons (Fig. 4E) [35]. Whereas neither the expression level or distribution Nogo-A differs between P26 and P40 [15], [35], the fraction of Nogo-A associated with myelin-membranes does not appear appropriately positioned to inhibit dendritic spine dynamics in layer I of barrel cortex.

**Figure 6:**
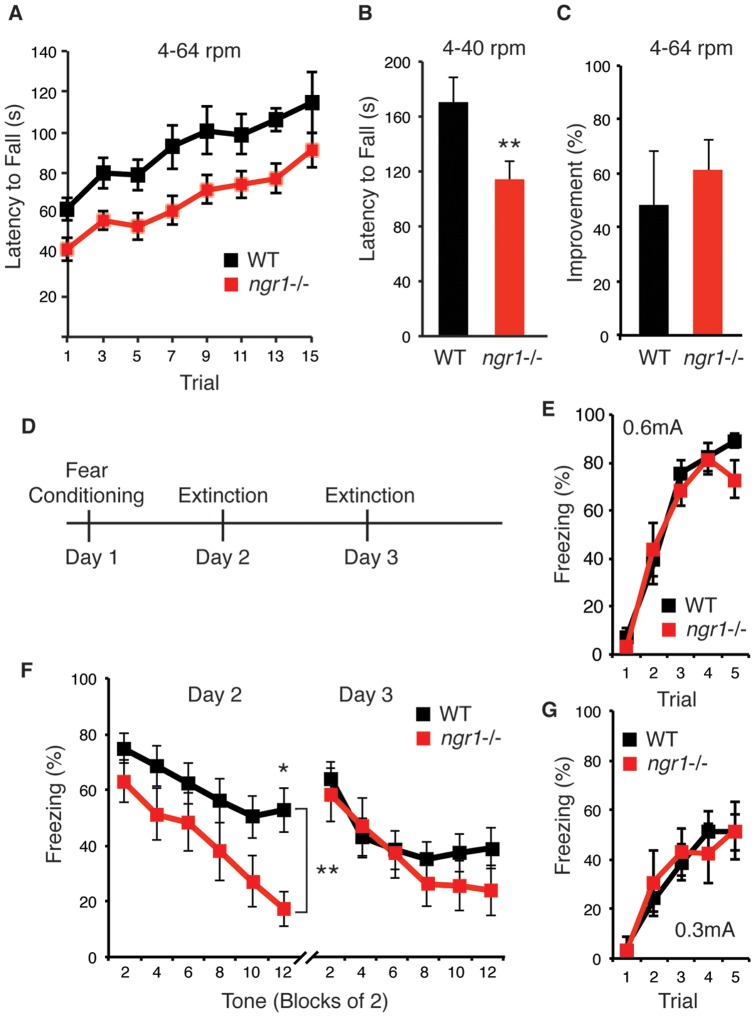
Motor learning is normal in NgR1 mutant mice but consolidation of fear extinction is impaired. (A) *Ngr1* mutant mice display a deficit in overall performance on the rotarod, but the rate of improvement is similar in WT and *ngr1−/−* mice (WT n = 8, *ngr1−/−* n = 9) (**, p<.01; *, p<.05). (B) *Ngr1* mutant mice also exhibit a mild deficit at a slower acceleration rate at the conclusion of training (p<.05, unpaired two-tailed t-test with Welch's correction). (C) The average percent improvement plotted as the percent difference in average latency to fall of the second two trials and last two trials. Improvement is similar between WT and *ngr1−/−* mice (p>.22, Kolmogorov-Smirnov test) (D) Schematic for fear conditioning and extinction protocol. On day 1, adult female mice were conditioned to an acoustic tone that co-terminated with a 1 second foot shock (0.6 mA). On days 2 and 3, mice were presented with 12 unpaired tones during a 30′ period. (E) Extinction of the fear response is plotted at percent time spent freezing averaged across two consecutive trials on day 2 and day 3 (n = 16 WT, n = 12 *ngr1−/−*). Extinction between the two genotypes differs across trials during day 2 but not day 3 by RM-ANOVA (bracket, day 2, p<.005; day 3, p>.42). (F) Acquisition of the freezing response is similar between WT and *ngr1−/−* mice on consecutive trials of the conditioned (tone) and unconditioned (0.6 mA shock) stimulus (n = 16 WT, n = 12 *ngr1−/−*). (G) Acquisition of the freezing response is similar between WT and *ngr1−/−* mice on consecutive trials of the conditioned (tone) and a milder unconditioned (0.3 mA shock) stimulus (WT, n = 8; *ngr1−/−*, n = 6).

As the basal dynamics of both dendritic spines and axonal boutons of *ngr1−/−* mice were indistinguishable from WT mice, next we explored if enhanced motor coordination might contribute to the better performance by *ngr1−/−* mice on the gap-cross assay. We examined the performance of *ngr1−/−* mice on the rotarod, a standard test of motor coordination. Two studies have identified that *ngr1−/−* mice exhibit a mild deficit in overall performance on the rotarod [20], [30]. However, a more recent study reported that conditional *ngr1flx/flx*; *Cre-ERT2* mice, in which the *ngr1* gene was deleted in adulthood by tamoxifen injection, have normal performance on the rotarod but a much faster rate of improvement [11]. We examined the overall capacity and improvement in rotarod performance for WT and *ngr1*−/− mice with a training protocol that employs a gradually acceleration of the rotating rod from 4 to 64 rpm [11]. *Ngr1*−/− mice exhibited a mild but statistically significant deficit in overall performance across all trials (WT, n = 7; *ngr1−/−*, n = 9; p<.02, two-way ANOVA) (Fig. 6A). In addition, we confirmed the relative magnitude of this deficit at the slower acceleration rate described in preceding studies (4–40 rpm) (p<.02) (Fig. 6B). WT and *ngr1*−/− mice also displayed a similar rate of improvement (WT, n = 7; *ngr1−/−*, n = 9; p>.19, Kolmogorov-Smirnov test) (Fig. 6C). In preliminary studies, we observe that *ngr1flx/flx; ER-Cre* mice following gene deletion by tamoxifen administration also improve on the rotarod similar to WT mice (data not shown).

To explore if *ngr1−/−* mice may have a deficit in fear induction or extinction, we tested *ngr1*−/− and WT mice with a tone-associated fear conditioning paradigm [10], [11], [31] (Fig. 6D). The establishment of the freezing response during conditioning was indistinguishable between WT and *ngr1*−/− mice (WT, n = 16; *ngr1−/−*, n = 12) (Fig. 6E). However, the extinction of the freezing response was significantly more pronounced in *ngr1*−/− mice during the first series of extinction trials on day 2 (WT, n = 16; *ngr1−/−*, n = 12; p<.005) (Fig. 6F), and the consolidation of this extinction was impaired in *ngr1*−/− mice as the freezing response rebounded on day 3. *Ngr1* mutant mice displayed extinction of freezing responses on day 3 similar to day 2 and WT mice on day 3 (p>.42) (Fig. 6F). As *ngr1*−/− mice exhibited a lower average freezing response for every trial on day 2, we re-examined their freezing response during the conditioning phase to determine whether NgR1 mutant mice possessed a deficit in tone-associated fear conditioning. As the current of the foot shock (0.6 mA) applied for tone-associated fear conditioning was higher than that required to generate a conditioned response, we tested conditioning with a foot shock at a lower current (0.3 mA) in a smaller cohort of mice (WT, n = 8; *ngr1−/−*, n = 6). We observe that WT and *ngr1*−/− mice also display similar tone-associated fear conditioning under these milder conditions (Fig. 6G).

## Discussion

The *ngr1* gene has been reported to limit the rate of motor learning and determine the set point for synaptic turnover in adult motor and sensory cortex [11]. To evaluate if *ngr1* restricts tactile performance or learning, we examined the performance and learning of *ngr1−/−* and WT mice with the gap cross assay. We observe that *ngr1−/−* mice perform significantly better on this task. However, this greater overall performance by *ngr1−/−* mice was not associated with a greater rate of learning at any gap distance across sessions. The percent improvement with experience averaged across 19 WT mice and 14 *ngr1−/−* mice was similar at both ‘nose’ and ‘whisker’ distances despite significantly better initial performance by *ngr1−/−* mice.

To determine if this elevated performance at ‘whisker’ distances might arise from greater motor coordination, we evaluated the performance of *ngr1−/−* and WT mice on the rotarod. Similar to preceding publications [11], [20], [30], we observe that *ngr1*−/− mice exhibit a mild deficit in performance. Despite this lower overall performance, *ngr1*−/− mice improve over subsequent trials similar to WT mice. In addition, we confirmed that *ngr1*−/− mice demonstrate the reported deficit at the slower acceleration rate published by Kim et al. (2004) and Lee et al. (2004).

We also considered if *ngr1−/−* mutant mice exhibit aberrant fear induction that would correlate with the greater performance on the gap-cross assay. To explore this possibility, we examined the induction of the freezing response in a tone-associated fear-conditioning paradigm. However, the rate of induction and the overall magnitude of the freezing response were indistinguishable between *ngr1−/−* and WT mice at a range of intensities for unconditioned stimulus. In addition, *ngr1−/−* mice display less time near the margin in an open field activity monitoring enclosure [20], consistent with an increase in anxiety in a novel environment.

We also evaluated the extinction of tone-associated fear conditioning in WT and *ngr1*−/− mice, another paradigm of experience-dependent learning. We observe that female *ngr1*−/− mice display greater extinction of the freezing response on day 2 but this extinction is not consolidated on day 3. This phenotype is opposite to that reported for male *ngr1*−/− mice previously [11]. Modest gender differences have been reported in some mouse behavior and learning paradigms [36]. However, fear conditioning and extinction learning are largely resistant to gender, and the effects of gonadal hormones are distinct from the differences in the pattern of fear induction, extinction, and consolidation that we observe between WT and *ngr1−/−* female mice [37], [38]. As induction and extinction of the freezing response in control mice are similar between the two studies (and genders), are robust, and are consistent with a preceding published report [31], differences in details of the design and/or execution of the experimental protocol are unlikely to result in the opposing patterns of extinction observed with the *ngr1−/−* mice. Interestingly, this rebound of the freezing response after extinction may be an adverse consequence of greater cortical plasticity. Perhaps the mechanisms that permit faster extinction of the freezing response in *ngr1−/−* mice also limit the consolidation of this experience-dependent behavioural adaptation. Additional studies will be required to elucidate the potential role of *ngr1* in extinction and consolidation of tone-associated fear conditioning.

Whether the behavioral phenotypes of *ngr1* mutant mice are a consequence of deletion of the gene within neocortex is not yet known. NgR1 is also expressed in thalamus, hippocampus, and amygdala [39]. Loss of *ngr1* may affect the circuitry or plasticity in these brain structures to alter the performance of mice on the gap-cross, rotarod, and fear-conditioning learning paradigms. Future experiments combining the *ngr1flx* allele with transgenic expression of cre recombinase restricted to cortical neurons or subcortical neurons may be able to answer this question [40], [41].

One model posits that the onset of myelination in cortex presents the Nogo-A ligand expressed by oligodendrocytes to signal through NgR1 to restrict cortical spine dynamics [11]. In support of this model, an increase in the distribution of myelinated fibers in visual cortex coincides with the closure of the critical period for developmental ocular dominance plasticity [15]. However, the onset of myelination does not coincide with the decrease in spine dynamics observed in the transition from late adolescence to adulthood elsewhere in cortex. Myelination is extensive in barrel cortex by P26 when spine dynamics are greater in layer I [34]. By P40, when spine turnover is decreasing to stable adult levels, myelination is more extensive in layer II/III, but remains sparse in layer I. Thus, myelinated axons do not appear appropriately positioned to regulate cortical spine dynamics in layer I of S1 barrel cortex. Furthermore, the distribution of Nogo-A expression is different than the pattern of myelination. Nogo-A is expressed by most neurons in adult neocortex although stronger expression is observed in putative oligodendrocytes [35]. This expression pattern does not change appreciably from P26 to P40. In V1, immunoblotting reveals that Nogo-A levels are comparable from P20 to P60 [15]. The topology of Nogo-A, a member of the reticulon family of resident endoplasmic reticulum proteins, is also controversial [42]–[44]. Thus, how oligodendritic Nogo-A might inhibit dendritic spine turnover and stability at P40 but not P26 is obscure as the distribution and expression of the protein appear unchanged. The roles of neuronal Nogo-A are largely unknown.

Whisker-dependent learning, motor learning, and fear conditioning are all associated with elevated cortical spine dynamics in layer I [9], [10], [45], [46]. These *in vivo* imaging studies, as well as the experiments presented here, examine dendritic spines of the apical tufts of only a small percentage of layer V pyramidal neurons, and in some cases, layer II/III pyramidal neurons, within the cortical column. Axons traversing layer I include thalamocortical axons, intracortical axons, and collaterals of local pyramidal neurons [29], [47]. The subpopulation of pyramidal neurons expressing fluorescent protein in these transgenic mice seem likely to represent the structural synaptic plasticity in layer I (but see below). Yet whether alterations to anatomical connectivity in layer I observed during learning influences cortical function directly, or reflects coincident plasticity elsewhere within cortical circuitry, is unclear.

We observe that *ngr1* mutant mice display normal cortical spine turnover. A recent study by Akbik et al. reported that *ngr1* mutant mice display dramatically elevated cortical spine turnover [11]. The reasons for the differences between our results and those presented in the study Akbik et al. are unclear. There are important and extensive similarities between the experiments presented here and those published by Akbik et al. Both studies employ the same strains of *ngr1* mutant mice. The two studies measure dendritic spine and axonal bouton turnover in the same regions of sensory cortex in mice of similar ages. The two studies employ the same EGFP-M transgene to sparsely express GFP in cortical neurons in similar experiments. Yet despite these similarities in experimental design, we were unable to reproduce the central findings that *ngr1*−/− mice display dramatically elevated turnover of dendritic spine or axon boutons under normal conditions. Whether *ngr1* mutant mice display greater synaptic structural plasticity during sensory adaptation or learning remains to be determined.

However, there are some differences in the imaging experiments between the two studies. We employed cranial windows to image dendrites and axons repeatedly at multiple consecutive 4-day intervals in regions of sensory cortex. We confirmed that the windows were properly positioned in a subset of mice with optical imaging of intrinsic signals. Akbik et al used predominantly the ‘thinned-skull’ transcranial technique to image dendrites and axons from YFP-H transgenic mice at either a 2-day interval or a 14-day interval near specified stereotaxic coordinates. However, they also imaged the identical strain of *ngr1−/−* mice expressing GFP from the same EGFP-M transgene through cranial windows as we employed. They concluded that these two preparations yield similar results. Thus, a core set of imaging experiments were performed under conditions that are directly comparable. We did not observe that dendritic spine and axonal bouton turnover in *ngr1*−/− mice are twice that of WT mice as reported.

Transcranial imaging and cranial window imaging have distinct advantages and disadvantages [24], [48]. The surgical preparation for ‘thin skull’ imaging is faster, more reliable, and unlikely to damage the underlying cortex. However, the small cortical region accessible for imaging often requires employing YFP-H transgenic mice that express fluorescent protein in many more neurons relative to the comparable EGFP-M transgenic mice [22], [24]. This broader expression often results in a dense lattice of labeled dendritic arbors in layer I, especially in older mice. With this approach, typically 100–150 spines are imaged from many distinct segments of dendrite originating from a number of neurons across often a single 2-day or 14-day interval. By comparison, the surgical preparation for cranial windows is more elaborate, the rate of successful surgery lower, and the consequence of inflammation or damage to the region of interest are a concern. However, the imaging region is significantly larger and compatible with the more sparse EGFP-M transgenic line that offers greater imaging contrast. In addition, this stable preparation permits repeated imaging of the same dendritic structures over extended periods of time. Thus, transcranial imaging may provide a more general sampling of cortical synaptic structural dynamics because spines are distributed among multiple neurons, whereas cranial window imaging permits a more specific analysis of individual neurons. On average, Akbik et al. imaged approximately 100 spines per mouse for one time interval for both transcranial and cranial window imaging. By comparison, we imaged on average 300 spines per mouse over three consecutive time intervals.

Repeated imaging improves the statistical power and precision of estimates of spine turnover and stability relative to imaging a single time interval. Although some controversy persists regarding the relative magnitude of dendritic spine turnover observed by transcranial imaging [48], [49] versus cranial window imaging [6], [24], several groups have both independently and collectively verified that cortical spine dynamics imaged through cranial windows are consistent for weeks to months [1], [3], [8], [24]. Notably, a recent study monitored the spine stability and density by imaging the same dendrites of layer V neurons in S1 through cranial windows in adult mice for more than a year [50]. Statistical analyses of the imaging data presented in this study reveals that we would have detected a 25% change in spine turnover as significant (p<.05). Akbik et al. report in similar experiments that spine turnover in *ngr1*−/− mice is twice that of WT mice, the largest increase in spine dynamics reported for age-matched mice for any genetic, pharmacologic, or environmental manipulation.

Independent of the imaging approach, high quality images of dendritic spines are paramount for accurate analysis of spine turnover and stability. We present several examples of the quality of images of dendritic spines collected in our study (Figures 2 and 3). These images are ‘best projections’, a montage of the best focal plane for each spine within a stack of images in the *z*-plane. This montage then receives only linear contrast adjustment, save for the high magnification images in panel 3B that also were filtered with a Gaussian blur for presentation. These images reflect the resolution and contrast of the *z*-stacks of raw images we examined for analysis. We consider these images suitable for identifying dendritic spines.

The YFP-H transgene exacerbates age-related axon pathology in some regions of the CNS [51]. As much of the imaging by Akbik et al was performed in YFP-H mice 6 months of age, near the age at which is the appearance of axonal spheroids is apparent, perhaps *ngr1* inhibits cortical anatomical plasticity in response to similar proximal or distal disruptions in axonal structure. Future studies will be required to ascertain if an unknown difference in imaging technique, spine analysis methodology, or difference between the EGFP-M and YFP-H transgenes contributes to the opposite findings of these two studies.

Overall, we conclude that while *ngr1* regulates tactile and motor task performance, it does not limit the rate of tactile or motor learning nor determine the low set point for synaptic turnover in adult sensory cortex.
